# Discovering co-occurring patterns and their biological significance in protein families

**DOI:** 10.1186/1471-2105-15-S12-S2

**Published:** 2014-11-06

**Authors:** En-Shiun Annie Lee, Sanderz Fung, Ho-Yin Sze-To, Andrew K C Wong

**Affiliations:** 1 Department of Systems Design Engineering, University of Waterloo, N2L 3G1 Waterloo, Canada; 2 Computer Science and Engineering, Chinese University of Hong Kong, Shatin, Hong Kong

**Keywords:** Spectral Clustering, Align Pattern Clustering, Jaccard Index, Ubiquitin, Cytochrome C, Protein Function Prediction, Sequence Patterns

## Abstract

**Background:**

The large influx of biological sequences poses the importance of identifying and correlating conserved regions in homologous sequences to acquire valuable biological knowledge. These conserved regions contain statistically significant residue associations as sequence patterns. Thus, patterns from two conserved regions co-occurring frequently on the same sequences are inferred to have joint functionality. A method for finding conserved regions in protein families with frequent co-occurrence patterns is proposed. The biological significance of the discovered clusters of conserved regions with co-occurrences patterns can be validated by their three-dimensional closeness of amino acids and the biological functionality found in those regions as supported by published work.

**Methods:**

Using existing algorithms, we discovered statistically significant amino acid associations as sequence patterns. We then aligned and clustered them into Aligned Pattern Clusters (APCs) corresponding to conserved regions with amino acid conservation and variation. When one APC frequently co-occured with another APC, the two APCs have high co-occurrence. We then clustered APCs with high co-occurrence into what we refer to as Co-occurrence APC Clusters (Co-occurrence Clusters).

**Results:**

Our results show that for Co-occurrence Clusters, the three-dimensional distance between their amino acids is closer than average amino acid distances. For the Co-occurrence Clusters of the ubiquitin and the cytochrome c families, we observed biological significance among the residing amino acids of the APCs within the same cluster. In ubiquitin, the residues are responsible for ubiquitination as well as conventional and unconventional ubiquitin-bindings. In cytochrome c, amino acids in the first co-occurrence cluster contribute to binding of other proteins in the electron transport chain, and amino acids in the second co-occurrence cluster contribute to the stability of the axial heme ligand.

**Conclusions:**

Thus, our co-occurrence clustering algorithm can efficiently find and rank conserved regions that contain patterns that frequently co-occurring on the same proteins. Co-occurring patterns are biologically significant due to their three-dimensional closeness and other evidences reported in literature. These results play an important role in drug discovery as biologists can quickly identify the target for drugs to conduct detailed preclinical studies.

## Background

Identifying functional regions on proteins is essential for understanding biological mechanisms and for designing new drugs. Due to the accessibility to protein sequences on the web, it is more effective to look for conserved segments from a set of functionally similar protein sequences than to perform laborious and time-consuming experiments and computationally intensive modeling. The study of conserved functional regions relies on the assumption that amino acids in functional regions are integral and thus undergo fewer mutations throughout evolution than less functionally important amino acids [1]. Therefore, the functional regions of protein structures can be obtained from analyzing protein sequences that have similar biological functions.

Multiple sequence alignment (MSA) [2,3] is a traditional computational method which is capable of aligning homologous protein sequences that are highly similar. However, it is unable to discover functional regions in more divergent protein sequences. Consequently, MSA is a global alignment method suitable for studying closely related proteins but not proteins that have only region-wise, partially functional similarities [4]. It has also been shown that finding the global optimal alignment is an NP-complete problem [5]. Coupling analysis [6-8] is a method based on MSA that examines the substitution correlation between two aligned columns within the MSA. This study hypothesizes that if two residues form a contact within a protein, then an amino acid substitution at one position is expected to be compensated for by a substitution in another position over the evolutionary time-scale. This observation suggests that co-occurring residues on the same protein can provide insight into the protein's structure. However, due to the dependence on MSA and the complexity of the method, determining the underlying statistical model requires a large number of homologous non-redundant protein sequences. Evolutionary tracing [1] is another method based on clustering alignments. The consensus within and across each group is identified to allow the study of divergent residues that are globally or functionally preserved in a protein family. Once again, evolutionary tracing is based on full sequence similarity requiring mutagenesis information for clustering [9]. Hence, it is not effective for revealing local functionality. Both coupling analysis and evolutionary tracing are based on examining pairwise amino acid correlations from MSA which focuses on two identified sites and does not take into account other sequence information.

In comparison to traditional methods, our algorithm finds and analyzes higher order sequence patterns in conserved regions, improving the capacity to reveal cross pattern association and local and distant functionality. In our previous work, we introduced Aligned Pattern Clusters (APCs) [10] to represent functional regions as an alternative to position weight matrices [11]. Aligned Pattern Clusters are sequence patterns with variations and conservation without assuming independence between residues [10] at sites. Its strength lies in the retention of statistical significance along the amino acids on a sequence and also the tracking of distribution of their occurrences across the sequences. With this novel representation, we are now able to exploit the APC occurrences and study the co-occurrence between their patterns on the same protein sequence.

We hypothesize that co-occurring patterns reflect the joint functionality that are needed for co-operative biological functions such as chemical bonds or binding sites. Thus, we address the following two research questions: 1) Given a set of homologous protein sequences, how can frequently co-occurring patterns be efficiently discovered? 2) How can the biological reasoning and significance of these co-occurrences be confirmed? To test these hypotheses, we used our co-occurrence clustering algorithm to find highly co-occurring patterns among a cluster of APCs and then studied their biological functions. First, we collect homologous protein sequences from the protein databases Pfam [12] and UniProt [13] as input. Next, we design an efficient algorithm based on our previous work [14,10] to find and represent the frequently co-occurring patterns. Finally, we verify our results by comparing the three-dimensional distance between the co-occurring patterns against the average distance between the regions spanned by the patterns. To confirm the biological functions of the co-occurrences, we search the related scientific literature to support the conceived role of these co-occurring patterns.

In view of the above mentioned computational results and biological observations accomplished in this paper, the contributions of this study mirror the answers to the research questions in two ways. First, we have established an algorithm that discovers co-occurring functional regions that are statistically reliable, measurable, and efficient. To our knowledge, this study is the first to identify the co-occurrence of patterns rather than residues. Compared to existing algorithms used to study correlations in amino acid residues, the novelty of our algorithm is that it does not require a large number of homologous protein sequences to identify pattern co-occurrences. Secondly, we have verified these co-occurrences by using the co-occurring patterns' three-dimensional closeness and by searching biological literature for support, enriching our understanding of the underlying mechanism. Novel co-occurrence relationships will provide new insight for the biological community for use in their study on protein functionalities.

## Methods

### Algorithm definition and details

**Figure 1:**
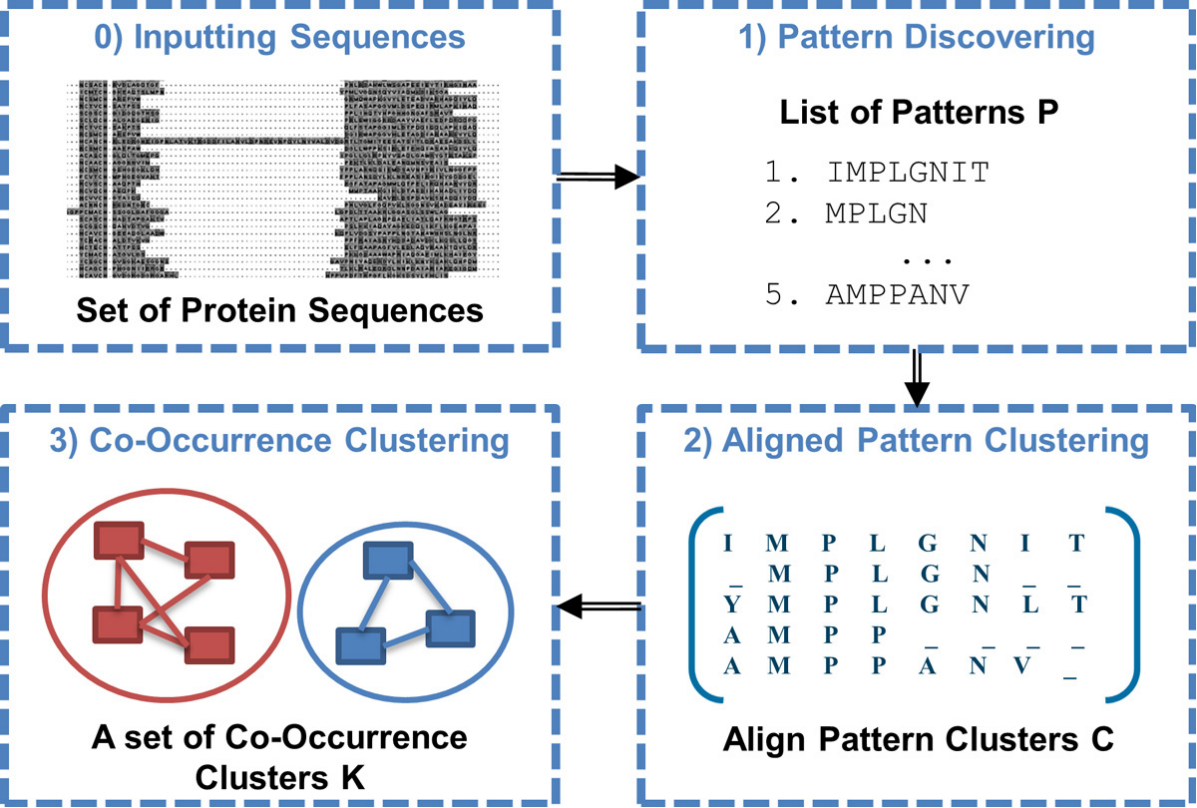
**The overall process of our methodology is represented by a pipeline consisting of three algorithms**. 0) the input is a set of sequences from the same protein family; 1) the published pattern discovery algorithm, which results in a list of patterns; 2) the published APC algorithm, which results in a set of APCs; and 3) the new Co-Occurrence Cluster algorithm, which cluster APCs by their co-occurrence scores.

The methodology proposed in this paper combines three algorithms together to obtain the Co-occurrence Cluster of Aligned Pattern Clusters (Co-occurrence Cluster) (Figure 1). The first two algorithms are adopted from our previously published research: 1) a pattern discovery algorithm that discovers statistically significant sequence patterns from a set of sequences of a protein family while pruning the redundant patterns [14]; 2) an Aligned Pattern Cluster (APC) algorithm that obtains compact aligned groups of statistically significant patterns referred to as APCs. These APCs contain variations with adjustable low information entropy [10]. Finally, in the third and main contribution algorithm of this paper, Co-occurrence Clusters are obtained by clustering the APCs discovered using spectral clustering [15] with a co-occurrence score adopted as a measure of distance.

#### Input data [16]

"Let Σ be the protein alphabet containing twenty standard amino acids $\left\{\sigma_{1},\sigma_{2},\;\ldots \;,\sigma_{|\sum |-1,}\sigma_{\left|\sum \right|}\right\}$. A protein sequence, $s=s_{1}s_{2}\;\ldots \;s_{|s|-1}s_{\left|s\right|}$, is represented by consecutive amino acids from the alphabet Σ, where each $s_{i}\in \sum$ and *s *is of length |s|." [16] The input dataset is a set of protein sequences from the same protein family. Sequence patterns are then discovered from this input dataset in the next step.

#### Pattern discovery [16]

"Sequence patterns with statistically significant amino acid associations are first discovered [14]. They are defined as an ordered sequence of interdependent symbols *p *= *s*^1^*s*^2^*...s^n ^*from the alphabet Σ. The pattern *p *has length *n*, and the *i^th ^*symbol that appears in the sequence is *s^i^*. The list of patterns resulting from the pattern discovery algorithm is represented by $\mathbb{P}=\left\{p^{i}|i=1,...,|\mathbb{P}|\right\}=\left\{p^{1},p^{2},...,p^{|\mathbb{P}|-1},p^{\left|\mathbb{P}\right|}\right\}$

and are pruned of redundant patterns." [16]

#### Aligned Pattern Clustering [16]

"An APC describes a set of sequence patterns that have been grouped due to their aligned similarities (as defined in [10]). Aligned patterns add gaps and wildcards to maximize the vertical similarity of amino acids between the patterns. Let an APC be defined as

(1) $$C^{l}=ALIGN\left(\begin{array}{c} p^{1}\\ p^{2}\\ \vdots \\ p^{m} \end{array}\right),$$

(2) $$={\left(\begin{array}{cccc} s_{1}^{1} & s_{2}^{1} & \cdots \; & s_{1}^{n}\\ s_{2}^{1} & s_{2}^{2} & \cdots \; & s_{n}^{2}\\ \vdots & \vdots & \vdots & \vdots \\ s_{1}^{m} & s_{2}^{m} & \cdots \; & s_{n}^{m} \end{array}\right)}_{m\times n},$$

where $s_{j}^{i}\in \sum \cup \left\{-\right\}\cup \left\{*\right\}$ is a symbol in pattern *pi *with a new column index *j*. Each of the $|{\mathbb{P}}^{l}|=m$ patterns in the rows of *Cl *is of length $|C^{l}|=n$." [16] Let a set of APCs be defined as $\mathbb{C}=\left\{C^{l}|l=1,...,|\mathbb{C}|\right\}=\left\{C^{1},C^{2},...,C^{|\mathbb{C}|-1},C^{\left|\mathbb{C}\right|}\right\}$.

#### Clustering APCs to Co-occurrence Clusters

Co-existence of patterns in different locations of the same protein may indicate that they are functionally related and important for the protein family. In Co-occurrence Clusters, we first apply a spectral clustering algorithm to cluster APCs using a co-occurrence score between APCs as the similarity measure. Let the graph *G *= (*V, E*) be a relationship graph with APCs as vertices. Let each vertex *v *be an APC, and let each weighted edge *e *be the co-occurrence for two APCs; the edge weight is the co-occurrence score to be defined later between the two APCs. The spectral clustering algorithm is used to obtain Co-occurrence Clusters based on the co-occurrences between the APCs.

*Co-occurrence score *[16] To tell how many patterns out of the total number of the discovered patterns co-occur in two APCs, we need a co-occurrence score which will be used as the similarity measure for clustering co-occurrent APCs. "The co-occurrence scores quantify how often patterns in two APCs appear together on the same sequence. The Jaccard index is adopted [17]:

$$J=\frac{\left|C_{seq}^{1}\cap C_{seq}^{2}\right|}{\left|C_{seq}^{1}\cup C_{seq}^{2}\right|},$$

where $C_{seq}^{1}$ = sequences that contain patterns from APC *C*^1 ^and $C_{seq}^{2}$ = sequences that contain patterns from APC *C*^2 ^." [16]

The APC pairs are ranked by co-occurrence score and listed in descending order. When two or more APC pairs have the same score, the sequence count of the union of the two APCs $\left(\left|C_{seq}^{1}\cup C_{seq}^{2}\right|\right)$ is used as a secondary ranking criteria, i.e., the pair with a higher union size indicates that it covers more sequences and, hence, should be ranked higher.

*Spectral clustering *For spectral clustering [15], an adjacency matrix *W *is first created and filled with the co-occurrence score between the APCs. Let *W *be an *n *by *n *matrix (*n *is the vertex count in *G*), where *W *(*i, j*) is the adjacency weight between vertex *v_i _*and *v_j _*, i.e., the co-occurrence score between vertex *v_i _*and *v_j _*. The following matrices was first constructed:

$$d_{i}=\underset{j}{\sum }W\left(i,j\right).$$

$$D=diag\left(d_{1},...,d_{n}\right),$$

where *D *is an *n *by *n *matrix.

Next, using the adjacency matrix, a Laplacian matrix *L *is created, and *L*'s eigen-vectors are calculated. Using random walk, construct the Laplacian matrix

$$L_{rw}=I-D^{-1}W$$

where *I *is an *n *by *n *identity matrix. Find both the eigenvalues and their corresponding eigenvectors for *Lrw *and sort the eigenvectors by the ascending order of their eigenvalues.

Finally, the eigenvectors are then used as positions for the APC vertices *v*, with the weighted edges *e *being the Euclidean distance between *v *in the vertex space of *G *and its neighbours. K-means clustering is applied to *G*, minimizing the Euclidean distance of the eigenvectors between the vertices. Let *k *be the final cluster count, defined as the count before the largest difference between consecutive eigenvalues [15]. We use the first *k *columns in the eigenvectors for clustering. Each row in the eigenvector corresponds to an APC vector, with each vector having *k *values. Together the row and columns make a point in *k*-dimensional space. Apply the k-means clustering algorithm on these given *k *points, but instead of maximizing similarities between the points within clusters, minimize the distances between the points.

*Comparison of clustering algorithms *Two other clustering algorithms are implemented to compare with spectral clustering: that is, the k-means clustering and the hierarchical clustering.

A special variation of the k-means clustering algorithm called k-medoids [18] is used in this paper. APCs are used to represent the centroids since calculating a centroid with only co-occurrence scores between APCs is difficult. The medoids are initialized to be the first APC for each connected component due to the small number of APCs considered. During the clustering process, the medoids are updated by finding the APC that maximizes the co-occurrence score between itself and all the other APCs in the same cluster. Finally, to ensure that clustering provides the best possible results, five clustering indicators are computed to determine the optimal final number of clusters, i.e., optimum *k*, to be adopted for the k-medoids.

**Algorithm 1 **Spectral clustering

**Input: **A set of APCs  ℂ, adjacency matrix *W *, and the final number of clusters required by the final k-means clustering algorithm

**Output: **APC Clusters *K*_1_*...K_k_*

**for ***i *= 1 to |ℂ|**do**

$d_{i}=\sum_{j}w\left(i,j\right)$

end for

*D *= *diag*(*d*_1_*,..., dn*)

Let *I *be a |ℂ|x|ℂ| identity matrix

$L_{rw}=I-D^{-1}W$

Calculate the eigenvectors and their corresponding eigenvalues of *L_rw_*

Sort the eigenvectors by their increasing eigenvalues

Take the first *k *columns of eigenvectors

Let each row of the eigenvector represent an APC, and let each eigenvector column a dimension

Construct a *k*-dimension graph *G_k _*with the eigenvector values

Apply k-means clustering on *G_k _*, minimizing the Euclidean distance between the points within the clusters.

**return **{*K*_1_...*K_k_*}

The hierarchical clustering algorithm uses a maximum spanning tree (MST) with minimal cut. First, an MST is built using Prim's algorithm. Next, the minimal weighted edge of the MST is cut to separate the vertices, which are APCs, into two co-occurrence clusters. The second step is repeated until an optimal solution is achieved.

The runtimes to find the optimal solutions for the three clustering algorithms are as follows: *O*(*n*^4^) for hierarchical clustering, *O*(*n*^3^) for spectral clustering, and *O*(*n*^3^) for k-medoids clustering. During the edge-cutting phase for hierarchical clustering the algorithm must evaluate all possible MST edges, a maximum of *n *edges, with each edge taking *O*(*n*^2^). Since there are a maximum of *n *MST edges to cut, the total running time is *O*(*n*^4^). K-medoids clustering takes *O*(*n*^2^) only if the cluster count is given. However, the algorithm is run *n *times to compare and obtain the optimal cluster count for the optimal clustering solution. Hence, the optimal solution has a runtime of *O*(*n*^3^). In comparison, spectral clustering takes *O*(*n*^3^) even with cluster count given, as the matrix multiplication that occurs when calculating the Laplacian matrix takes *O*(*n*^3^). However, the matrix is calculated only once, the optimal cluster count is obtained through the eigenvalues, and the algorithm uses the same that for the k-medoids algorithm to find the optimal cluster. Hence, the total runtime for spectral clustering is the same as k-medoids clustering, *O*(*n*^3^). Because of the quicker runtime, spectral and k-means clustering are preferred over hierarchical clustering.

Moreover, the spectral clustering algorithm is selected over the k-means clustering algorithm used in [19] because of the nature of the data. Pfam [12] sequences are built from multiple sequence alignments with the help of hidden Markov model; thus, the sequences have been pre-processed for correctness. UniProt [13] sequences are collected from a string query search of the database, so the quality of the sequences depends on the search terms. Therefore, the sequence quality of UniProt is less consistent, making it unsuitable for clustering using the global centroid of k-means since the low-quality sequences are heavily affected by outliers [20]. Closest neighbour characteristic in the spectral clustering algorithm is beneficial in handling noisy data. Therefore, this algorithm was selected to cluster co-occurrent APCs.

#### Verification by three-dimensional structure

To evaluate the importance of the APC regions discovered, we use the three-dimensional distance between the protein segments corresponding to the APCs within the Co-occurrence Cluster. The rationale for using the three-dimensional distance is that if the APCs are close together in three-dimensional space then they will likely interact with one another. It thus provides biophysical support that these functional regions are of biological importance to the proteins in the protein family tested.

After applying Co-occurrence Clustering, we manually select the cluster that contains the lowest average eigenvector distance as the highly connected Co-occurrence Cluster. We relate these results to the corresponding three-dimensional protein structure from the Protein Data Bank (PDB) [21] using Chimera [22], highlighting the regions where the APCs, or parts of the APCs, appear. The distances between the APCs are calculated as follows: the positions of each carbon alpha in each APC region is averaged, creating an average centroid for each APC region. The Euclidean distance is then calculated amongst all centroids. Finally, the APC distance is compared to the average pairwise distance, which is the average Euclidean distance of all possible carbon alpha pairs in the structure.

Using only the highly connected Co-occurrence Cluster and finding its biological importance, we validate 1) that the co-occurrence score ranks important APC pairs over the less important one, 2) that co-occurrence clustering is able to separate the less important APCs out and 3) that our algorithm can provide reasonably good results in a timely manner, i.e. by not having to search through all APCs discovered.

### Datasets

The first dataset selected for our experiment contains two different protein families from UniProt, which are examined in subsequent detailed case studies. The first set is of ubiquitin protein sequences, downloaded on August 9th, 2012, with the following filters to obtain high quality sequences: having the name ubiquitin with a mnemonic starting with UB; and not containing the words ribosomal, modifier, factor, protein, conjugate, activating, or enzyme to remove other similar names. The second is of cytochrome c protein sequences, downloaded on December 20, 2013, similarly with the filters: having the name cytochrome c with the mnemonic CY*; not ending in "ase" to prevent the inclusion of oxidase or reducatase; and not containing biogenesis or probability to remove other similar names. Each sequence from UniProt has an organism name, which is next searched in UniProt Taxonomy to acquire the condensed taxonomy lineage. Finally, the top kingdom name is extracted as the class label.

Next, our method was run on the two UniProt datasets. For the 70 ubiquitin input sequences, the pattern-discovery step was executed with a minimal length of 5, a maximum length of 15, a minimum occurrence of 20, and a delta of 0.9 (for control of delta closed pattern pruning). The maximum length restricted long (or high order) patterns from being discovered in the highly conserved ubiquitin sequences. Aligned pattern clustering was then executed with the following settings: Global Alignment with Hamming Distance and heuristics conditions with a minimum consecutive column match of 3, a minimum conserved column of 1, and no relative position overlapping. For the 319 cytochrome c input sequences, the pattern discovery step was executed with a minimal length of 5, a minimum occurrence of 40, and a delta of 0.9. The increase in the minimum occurrence was due to the increase in the number of input sequences. Aligned pattern clustering was then executed with the same settings as above. Lastly, the co-occurrence score was computed, and the three clustering algorithms were run. For both datasets, spectral clustering and k-medoids resulted in producing the same Co-occurrence Cluster.

The second dataset contains nine different protein families downloaded from Pfam Release 3.2 for a large-scale study of the three-dimensional structure of proteins. Pfam was used due to its well curated and pre-processed data. The proteins are lipocalin [Pfam:PF000061]; bacterial rhodopsins [Pfam:PF00061]; bacterial antenna complex [Pfam:PF01036]; cytochrome c oxidase subunit I [Pfam:PF00115]; photo- synthetic reaction centre protein family [Pfam:PF00124]; leptin [Pfam:PF02024]; G-alpha subunit [Pfam:PF00503]; protein kinase domain [Pfam:PF00069]; and tyrosine kinase [Pfam:PF07714]. The pattern-discovery and the aligned pattern clustering steps were executed with the same settings as above, except the minimum occurrence, which was adjusted based on the number of sequences and their sequence similarity as listed in Pfam. After clustering, we picked the Co-occurrence Cluster with the lowest average eigenvector distance to be evaluated for the three-dimensional distance.

## Experimental results and discussions

### Proteins verified by three-dimensional structure

We applied our method to nine protein families, confirming that our algorithm is effective at finding important regions on any protein family. Table 1 displays the Co-occurrence Cluster of closely related APCs in the PDB structure of the related protein family. We found that these APCs are close in Euclidean distance in the three-dimensional space.

**Table 1:** Results from the nine protein families.

Protein name	Pfam ID	Co-occurrence cluster count	Size of the best cluster	PDB ID of the best cluster	Average APC distance of the best cluster	Average pairwise distance
Lipocalin	PF00061	6	4	2CZT	**16.77 Å**	19.26 Å
Bacterial rhodopsins	PF01036	2	2	1JGJ	**16.52 Å**	22.51 Å
Bacterial antenna complex	PF00556	4	5	1IJD	**0 Å**	19.92 Å
Cytochrome c oxidase subunit I	PF00115	2	25	3OM3	26.78 Å*	30.00 Å
Photosynthetic reaction centre protein family	PF00124	2	7	1PSS	**27.87 Å**	30.19 Å
Leptin	PF02024	2	14	1AX8	**15.73 Å**	18.37 Å
G-alpha subunit	PF00503	3	8	4G5O	**15.78 Å**	27.45 Å
Protein kinase domain	PF00069	2	2	3OZ6	**15.32 Å**	27.51 Å
Tyrosine kinase	PF07714	2	8	4HW7	**14.43 Å**	24.99 Å

Displays the Co-occurrence Cluster with the lowest average eigenvector distance, and are used to verify the algorithm's effectiveness with a PDB structure. The shorter distance in the comparison is bolded. * means that one or more APCs were not found.

**Figure 2:**
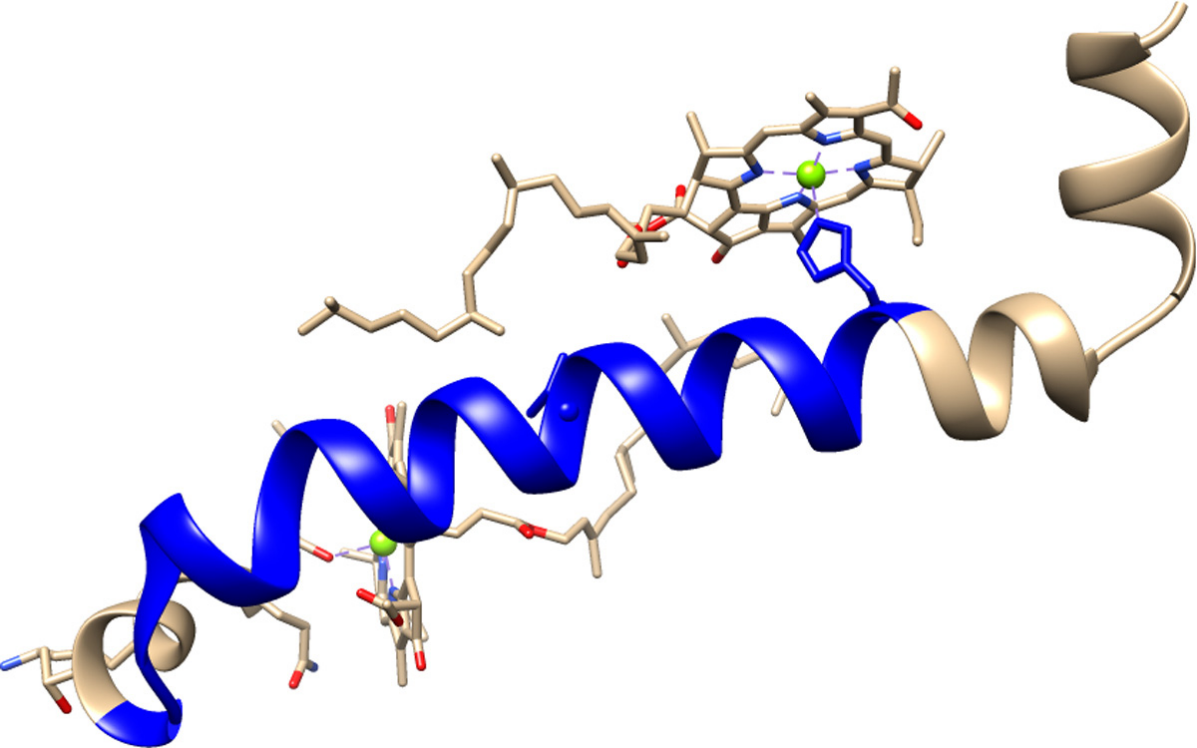
**Three-dimensional structure of bacterial antenna complex [PDB:1IJD]**. The set of all the patterns in the APCs in the Co-occurrence Clusterinspected are all contained within one continuous highlighted blue region, indicating how the APCs overlaps with one another.

Of interest are the results from the bacterial antenna complex family [Pfam:PF00556], where there is an average APC distance of 0 Å. The reason is that, despite having 5 APCs in the maximum co-occurrence cluster, all APCs overlap with one another, creating one long continuous region highlighted in blue (Figure 2). Furthermore, the highlighted region covers positions 9 to 31 of the structure, and has only 46 amino acids, i.e., the maximum co-occurrence cluster continuously covers close to half of the whole structure. The figure also indicates that [Pfam:PF00556] might be highly conserved, exhibiting only minor variations in its primary structure across different proteins in the family, especially in the regions covered by the maximum co-occurrence cluster. Another result where the maximum co-occurrence cluster covers most of the amino acids in the PDB structure is Leptin [Pfam:PF02024, PDB:1AX8], where only 14 amino acids are not covered by the APCs in the maximum co-occurrence cluster.

All the APCs within the cluster in all the experiments in Table 1 were closer in distance than the average pairwise distance, indicating a relation between co-occurring APCs and their distance in three-dimensional structures. We were able to observe some characteristics of the protein family, i.e., the conservation of its primary structure. Hence, our algorithm is proven to discover important conserved regions for protein families.

### Biological validation

In this section, we investigated the biological significance of Co-occurrence Clusters. Our experimental results revealed the Co-occurrence Clusters of ubiquitin and cytochrome c. Here we would like to study why co-occurring APCs are close to one another in spatial distance despite being far from each other in the primary sequence. Our hypothesis is that they need to form chemical bonding or co-operate in essential biological functions.

#### Ubiquitin case study

Ubiquitin (UBI) is a small (8.5kDa) protein that consists of a single polypeptide chain of 76 amino acids [23]. It plays an important role in ubiquitination, which is a post translational protein modification process where either a single ubiquitin or multiple chains of ubiquitin are attached to a substrate protein. To form a chain, a ubiquitin connects to another ubiquitin by binding the diglycine in its C-terminal tail to one of the seven lysine amino acids of its linking partner.

Ubiquitination is widely used in regulating cellular signaling [24]. It does so by allowing the attached ubiquitin in substrate proteins to be bound through proteins with ubiquitin-binding domains (UBD) [24]. Either attaching a ubiquitin to a target protein or connecting it to another ubiquitin is regulated by the sequential activity of ubiquitin-activating (E1), ubiquitin-conjugating (E2) and ubiquitin-ligating (E3) enzymes [24].

**Figure 3:**
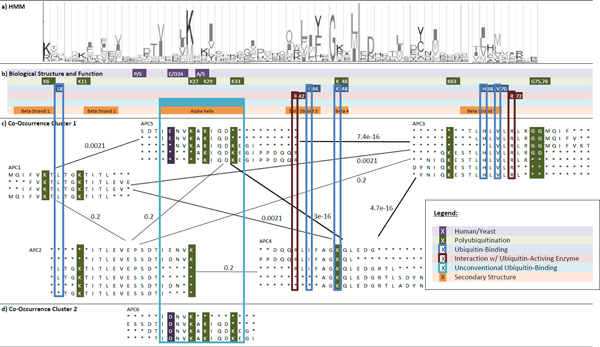
**Co-occurrence clusters of ubiquitin**. General Features: a) the top of the diagram is part of the HMM sequence profile of ubiquitin; b) the color shading blocks with legends immediately below mark the important amino acids and segments forming the important structure and function of the protein; c-d) the APCs discovered are represented by arrays of aligned amino acids; the color shaded columns correspond to the significant residues marked as in b); if the co-occurrences of patterns between APCs are frequent, the co-occurrence APCs are linked by an edge with weight representing co-occurrence score; treating APCs as vertices. A co-occurrence APC cluster is represented by a weighted graph linking co-occurring APC s; the important functional regions of the molecules as listed in Table 2 are highlighted in colored blocks specified by the legend. Specific Features: Note that APC 5 and APC 6 are not linked by co-occurrence since they belong to different taxonomical group and with different amino acids, Asp(D)24 and Glu(E)24, in the same column.

**Figure 4:**
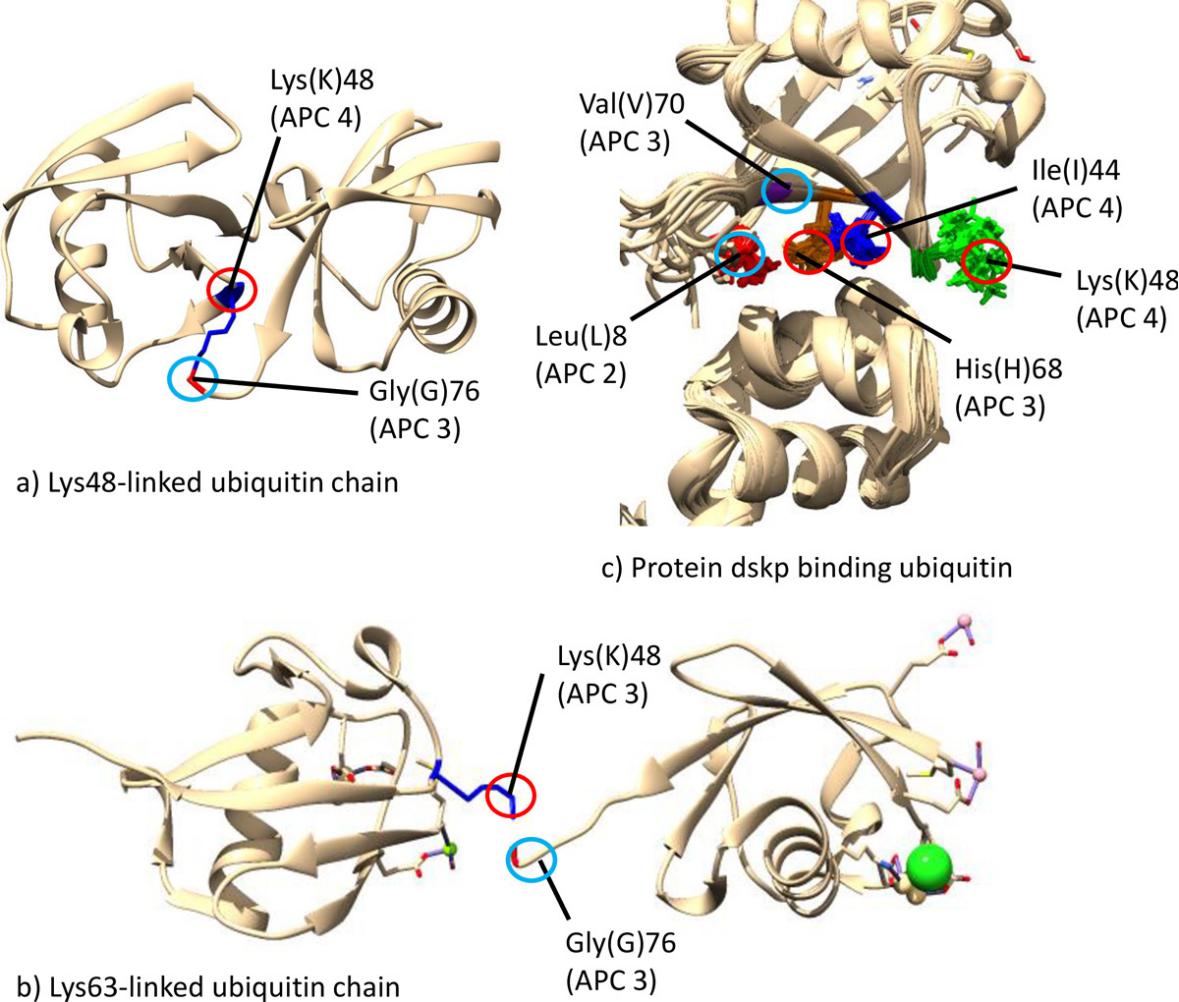
**Three-dimensional structures of ubiquitin [PDB:1AAR,2JF5,1WR1]**. The binding residues discussed in Table 2 and their functions are displayed. a) is the ubiquitin chain linked by the Lys(K)48 in APC 4 to the diglycine, b) is the ubiquitin chain linked by the Lys(K)63 in APC 4 to the diglycine, c) is the binding between dskp binding ubiquitin and ubiquitin by Leu(L)8 of APC 2, Val(V)70 of APC 3, Ile44(I) and Lys(K)48 of APC 4, and His(H)68 of APC 3.

When the seven lysine amino acids were mapped to our APCs, they were all covered (Table 2). According to the results of our co-occurrence clustering algorithm in Figure 3, the optimum number of cluster of the six APCs is two. The first cluster includes APC 1, 2, 3, 4 and 5; the second cluster includes APC 6 only. Their biological significance is discussed in Figure 4.

**Table 2:** Key residues covered by APC and their roles in the Co-occurrence Cluster 1 of ubiquitin

APC	Residue(s)	Role(s)	Literature
1	K6, K11	Lys(K)6 and Lys(K)11 are used for forming ubiquitin chain(s) in ubiquitination.	[23]
	L8	Leu(8) facilitates the interaction between ubiquitin and E1 enzymes.	[24,27]
2	K11, K27	Lys(K)11 and Lys(K)27 are used for forming ubiquitin chain(s) in ubiquitination.	[23]
	L8	Leu(8) facilitates the interaction between ubiquitin and E1 enzymes.	[24,27]
3	K63	Lys(K)63 is used for forming ubiquitin chain(s) in ubiquitination.	
	H68, V70	His(H)68 and Val(V)70 facilitate the binding between ubiquitin and ubiquitin-binding protiens.	[24,27]
	R72	Arg(R)72 facilitates the interaction between ubiquitin and E1 enzymes.	[25]
	G75,G76	Gly(G)75 and Gly(G)76 are are used for forming ubiquitin chain(s) in ubiquitination.	[23]
4	R42	Arg(R)42 facilitates the interaction between ubiquitin and E1 enzymes.	[25]
	I44	Ile(I) 44 is the binding site between ubiquitin and the ubiquitin-binding proteins.	[24,27]
	K48	Lys(K)48 is used for forming ubiquitin chain(s) in ubiquitination. It also facilitates the binding between ubiquitin and ubiquitin-binding proteins.	[23,24,27]
5	K27,K29,K33	Lys(K)27, Lys(K)29 and Lys(K)33 are used for forming ubiquitin chain(s) in ubiquitination.	[23]
	R42	Arg(R)42 facilitates the interaction between ubiquitin and E1 enzymes.	[25]

The APCs in the first cluster to co-occur for two reasons. First, each APC covers at least one Lysine (K). The diglycine in the C-terminal tail, i.e., Gly(G)75 and Gly(G)76 (green shade), is also covered in APC 3. As discussed, Lysine (K) and the diglycine in the C-terminal tail are both important for the formation of multiple ubiquitin chains. Both APC 5 and APC 3 also cover important residues for facilitating the interaction of ubiquitin with E1 enzymes [25]. Mutagenesis experiments demonstrated that the mutation of Arg(R)42 or Arg(R)72 (red blocks) destabilizes the binding between Ubiquitin and E1 enzymes significantly, thus in turn, destroying the biological functions of ubiquitin [25]. Second, all APCs except APC 5 cover the ubiquitin-binding residues. These residues are important for the tight binding of ubiquitin with ubiquitin-binding proteins [24]. Therefore, the APCs in the Co-occurrence Cluster 1 are due to both ubiquitination and ubiquitin-binding.

There is only one APC, APC 6, in the second cluster (Figure 3) which has no co-occurrence with other APCs. We also observed a certain degree of overlapping between APC 6 and APC 5. We propose two reasons to explain why APC 6 is not merged with APC 5 but exists alone in another cluster. First, the conserved amino acid in residue 24 of APC 6 and APC 5 is Asp(D)24 and Glu(E)24 (yellow shade), respectively. We found that ubiquitin of Viridiplantae (plant kingdom) has mostly Glu(E)24, whereas ubiquitin of Metezoa (animal kingdom) has mostly Asp(D)24 in our dataset, this site is also well-known for differentiating human (containing Glu(E)24) ubiquitin from yeast (containing Asp(D)24) ubiquitin [26]. Hence, APC 6 and APC 5 are not merged in this study, because they cover patterns with different amino acids in different species.

Second, APC 6 does not include ubiquitination-related Arg(R)42 and covers the alpha helix 1, from residues 23 to 34, more precisely than APC 5. Previous literature has discovered that alpha helix 1 is an unconventional recognition site of ubiquitin-binding proteins [27]. Experiments in the same study revealed that, even if Ile(I)44 and His(H)68 were mutated, a high affinity binding between protein CKS1 and ubiquitin would still be identified, thereby proving that ubiquitin is unconventionally bound by CKS1 [27]. It should be noted that the conventional and unconventional ubiquitin-binding is not mutually exclusive [27]. Hence, APC 5 in the first cluster and APC 6 in the second cluster are not merged. Where APC 5 represents the scenario that either only conventional ubiquitin-binding occurs or conventional and unconventional ubiquitin-binding co-occur, APC 6 represents the scenario that only unconventional ubiquitin-binding occurs. Our experimental results from ubiquitin and literature search give us very strong support for the biological significance of the discovered Co-occurrence Cluster.

#### Cytochrome c case study

Cytochrome c (cyt-c) is a small (12.4kDa), heme-containing protein that consists of approximately 104 amino acids [28]. It is an essential component of the electron transport chain in the mitochondria. The heme group of cyt-c accepts electrons from the complexes III (cytochrome b-c1 complex or cyt-bc_1_) and transfers electrons to the complexes IV (cytochrome c oxidase or cyt-c_1_) [28].

**Figure 5:**
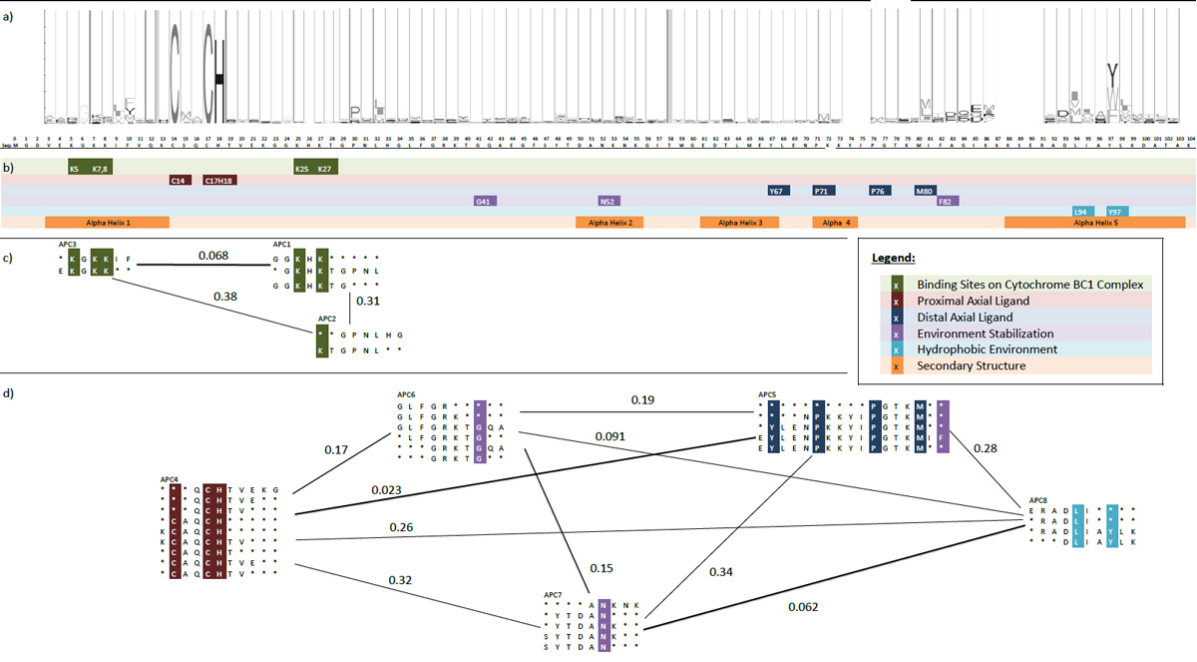
**Co-occurrence clusters of cytochrome c**. General Features is same as stated in Figure 3 c-d) Important functional regions as listed in Table 3 and 4, are highlighted here in color blocks as specified by the legend; Specific Features: Amino acids in Co-occurrence Cluster 1 facilitate the binding of cyc-1 on cyc-bc1 as listed in Table 3 and most of the amino acids in Co-Occurrence Cluster 2 are responsible for the stable axial ligand between cyc-t and the heme group.

According to the results of our co-occurrence clustering algorithm (Figure 5), the optimum number of clusters of the 8 APCs is 2. The first cluster includes APCs 1 to 3; the second cluster includes APCs 4 to 8. Their biological significance is discussed as below.

For the first cluster, we found that all the APCs covered residues that contributed significantly to the binding of cyc-1 on cyc-bc1. This is crucial for electron transfer. Experiments have established the importance of Lys(K)8, Lys(K)27 and, to a lesser extent, Lys(K)5, Lys(K)7, Lys(K)25 [29-31]. They are covered in the APCs in the first cluster (Table 3). Therefore, these APCs co-occur to facilitate the binding of cyc-1 on cyc-bc_1_.

**Table 3:** Key residues covered by APCs and their roles in co-occurrence cluster 1 of cytochrome c

APC	Residue(s)	Role(s)	Literature
1	Lys25, Lys27	The binding sites of cytochrome c cytochrome BC1 complex	[29-31]
2	Lys27	The binding sites of cytochrome c cytochrome BC1 complex	[29-31]
3	Lys5, Lys7, Lys8	The binding sites of cytochrome c cytochrome BC1 complex	[29-31]

**Figure 6:**
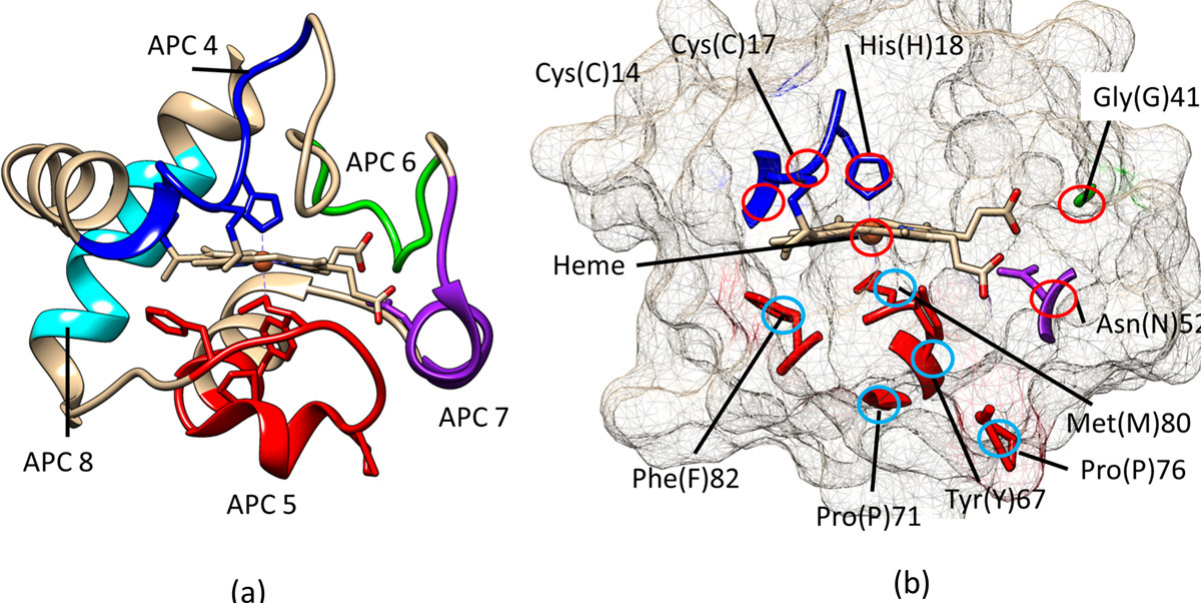
**Three-dimensional structure of cytochrome c [PDB:1HRC]**. a) The APCs in Co-occurrence Cluster2 as listed in Table 4. b) The amino acids from APCs in Co-occurrence Cluster2 mostly interact with the heme to stabilize the axial ligand, as confirmed by biological literature listed in Table 4.

For the second cluster, we found that all the APCs covered residues that were mostly responsible for the stable axial ligand between cyc-t and the heme group (Figure 6), which is the component that takes part in the redox reactions for the electron transfer between cyt-c and other complexes. APC 4 covered Cys(C)14 [32,33], Cys(C)17 [32,33] and His(H)18 [34,35]. His(H)18 [34,35] forms an axial ligand with the heme from the proximal front. Cys(C)14 [32,33] and Cys(C)17 [32,33] enhance and maintain the axial ligand between His18 and the heme. APC 5 covered Tyr(Y)67 [36,28], Pro(P)71 [37], and Pro(P)76 [38], Met(M)80 [35] and Phe(F)82 [39]. Met(M)80 [35] forms an axial ligand with the heme from the distal side. Tyr(Y)67 [36,28], Pro(P)71 [37], Pro(P)76 [38] stabilize and coordinate the axial ligand between Met(M)80 and the heme. Phe(F)82 [39] stabilizes the native heme environment. APC 6 covered Gly(G)41 [40], which holds the axial ligand between Met(M)80 and the heme. APC 7 covered Asn(N)52 [41,42], which maintains a hydrogen bond with the heme to stabilize the environment.

**Table 4:** Key residues covered by APCs and their roles in co-occurrence cluster 2 of cytochrome c

APC	Residue(s)	Role(s)	Literature
4	Cys(C)14	Cys(C) 14 enhances axial ligand strength between His18 and the heme.	[32,33]
	Cys(C)17	Cys(C) 17 enhances axial ligand strength between His18 and the heme.	[32,33]
	His(H)18	His(H)18 forms an axial ligand with the heme from the proximal front.	[34,35]
5	Tyr(Y)67	Tyr(Y)67, its hydroxyl group, forms a H-bond with side chains of Met80 for structural stabilization.	[36,28]
	Pro(P)71	Pro(P)71 helps coordinate the axial ligand between Met80 and the heme.	[37]
	Pro(P)76	Pro(P)76 helps coordinate the axial ligand between Met80 and the heme.	[38]
	Met(M)80	Met(M)80 forms an axial ligand with the heme from the distal side.	[34,35]
	Phe(F)82	Phe(F)82 helps stabilize the native heme environment.	[39]
6	Gly(G)41	Gly(G)41 helps stabilize the axial ligand between Met80 and the heme.	[40]
7	Asn(N)52	Asn(N)52 maintains a hydrogen bond with the heme to stabilize the environment.	[41,42]
8	Leu(L)94	One of Leu(L)94 or Tyr(Y)97 is required to provide a hydrophobic environment for the function of cyt-c.	[43]
	Tyr(Y)97	One of Leu(L)94 or Tyr(Y)97 is required to provide a hydrophobic environment for the function of cyt-c.	[43]

Although APC 8 did not cover any residues that are directly related to the axial ligands between cyt-c and the heme group, it covered residues that maintain the cyt-c structure. Among the 38 intra-molecular hydrophobic interactions reported in [41], APC 8 covered 17 (44.7%). It also covered Leu(L)94 [43] and Tyr(Y)97 [43], where one of them is required to provide a hydrophobic environment in order for cytc to function. Evidently, the APCs in the co-occurrence cluster 2 form and maintain stable axial ligands with the heme and also provide an appropriate structure and environment for cyt-c to function.

## Conclusions

In this paper, we address the two research questions that were first posed in the introduction. We answer the first research question on discovering co-occurrences by creating a novel algorithm that clusters APCs with frequently co-occurring patterns into an effective, statistical, and measurable Co-occurrence Clusters. We respond to the second research question on the biological significance of these Co-occurrence Clusters by their three-dimensional closeness and by biological functionality and structural integrity. We confirm that the Co-occurrence Cluster with the lowest average co-occurrence score is also closer in three-dimensional distance than the average amino acids in the three-dimensional structure. We also confirm that co-occurring APCs form chemical bonds or co-operate in essential biological functions as supported in biological literature. As a natural extension, we can use correlated amino acid variations to track evolutionary divergence and extend the algorithms to discover consistence and deviance of chemical properties. Since it is time-consuming to study the functional and structural sites for every target protein's drug interaction in detail, the ability to discover top-ranking Co-occurrence Clusters could also help to isolate the amino acids of biological significance. Hence, our method will have great potential to impact drug discovery and the biomedical community.

## List of abbreviations used

Aligned Pattern Cluster (APC), multiple sequence alighment (MSA), maximum spanning tree (MST), Protein Data Bank (PDB), ubiquitin (UBI), ubiquitin-binding domains (UBD), cytochrome c (cyt-c).

## Competing interests

The authors declare that they have no competing interests.

## Authors' contributions

EAL contributed to the idea, algorithm, experiments, and all aspects of the paper. SF contributed to programming the algorithm and running experiments. HYST contributed to the interpretation of biological literature. AKCW contributed to overall development of the research. All authors contributed to writing the paper.

## Declarations

Publication of this article has been funded by NSERC Discovery Grant (2450 500 105 0503 116642).

This article has been published as part of *BMC Bioinformatics *Volume 15 Supplement 12, 2014: Selected articles from the IEEE International Conference on Bioinformatics and Biomedicine (BIBM 2013): Bioinformatics. The full contents of the supplement are available online at http://www.biomedcentral.com/bmcbioinformatics/supplements/15/S12.
